# Characteristics and trends of antibiotic resistance in several gram-negative bacteria responsible for pneumonia in intensive care patients in Northern Vietnam, 2022-2024

**DOI:** 10.1016/j.ijregi.2025.100709

**Published:** 2025-07-18

**Authors:** Nguyen Quoc Phuong, Pham Ngoc Thach, Tran Van Giang

**Affiliations:** 1 Infectious Department, Hanoi Medical University, Hanoi, Vietnam; 2 Department of Intensive Care, National Hospital of Tropical Diseases, Hanoi, Vietnam

**Keywords:** Community-acquired pneumonia, Hospital-acquired pneumonia, Gram-negative bacteria, Antibiotic resistance

## Abstract

**Objectives:**

This study aimed to describe the features and trends in antibiotic resistance among gram-negative bacteria in pneumonia patients receiving intensive care in Northern Vietnam.

**Methods:**

Specimens were obtained from 222 patients in 2022-2024 and thereafter used for bacterial identification and antibiotic tests.

**Results:**

A total of 250 strains were identified from 222 specimens, including *Acinetobacter baumannii* (32.8%), *Pseudomonas aeruginosa* (23.6%), and *Klebsiella pneumoniae* (20.4%). Antibiotic sensitivity was higher in community-acquired pneumonia (CAP) than in hospital-acquired pneumonia (HAP). In CAP, the antibiotic sensitivity of *K. pneumoniae* was 36.4-90.9%, *P. aeruginosa* was 22.2-44.4%, and *A. baumannii* was 0-33.3%. However, in HAP, sensitivity decreased to 15.0-47.5%, 5.6-20.0%, and 1.9-12.9%, respectively. Three gram-negative bacteria in CAP showed lower resistance than HAP. Antibiotic resistance trends for *A. baumannii* and *K. pneumoniae* were steady, whereas *P. aeruginosa* rose to 20.4% in 2023 and 7.6% in 2024.

**Conclusions:**

Gram-negative bacteria were responsible for a significant proportion of pneumonia cases in intensive care patients. Antibiotic resistance in bacteria was lower in CAP than in HAP. The trend of developing antibiotic resistance in *A. baumannii* and *K. pneumoniae* remained stable, but *P. aeruginosa* rose from 2022 to 2024.

## Highlights

• Men in Vietnam have 2.77 times the rate of bacterial pneumonia as women.
• People over the age of 65 years are at equal risk for community-acquired and hospital-acquired pneumonia.
• *Acinetobacter baumannii, Pseudomonas aeruginosa*, and *Klebsiella pneumoniae* were the most prevalent bacterial causes of pneumonia.
• In community-acquired pneumonia, *K. pneumoniae* revealed a lower resistance rate than *A. baumannii* and *P. aeruginosa*.
• *K. pneumoniae* and *A. baumannii* showed no rise in resistance in 2022-2024, *P. aeruginosa* rose drastically.

## Introduction

Pneumonia, which includes community-acquired and hospital-acquired forms, is an acute infectious disease affecting the lung tissue, frequently seen in children under 5 years and adults aged over 65years [[1], [2], [3]]. It is a prevalent disease with the potential for severe complications because the average mortality rate ranges from 5% to 10%, particularly, among older individuals and those with preexisting comorbidities [[2], [3], [4]]. Currently, bacterial pneumonia remains the primary concern in lung diseases in Vietnam regarding occurrence and death rates [[5], [6], [7], [8]]. Nevertheless, the management of these infections has been negatively impacted by the rise and spread of antibiotic-resistant bacteria. In Vietnam, the primary pathogens responsible for community-acquired pneumonia (CAP) and hospital-acquired pneumonia (HAP) are mainly aerobic gram-negative bacteria, such as *Klebsiella pneumoniae, Enterobacter* spp., extended-spectrum β-lactamase (ESBL)–producing bacteria, *Pseudomonas aeruginosa*, and *Acinetobacter baumannii* [8,9]. Among these, *K. pneumoniae, A. baumannii*, and *P. aeruginosa* are particularly significant, exhibiting high resistance rates to multiple antibiotics, with their resistance continuing to grow, which reduces the effectiveness of treatments and raises mortality rates, especially with inappropriate antibiotic use [[10], [11], [12]].

The increased antibiotic resistance among gram-negative bacteria responsible for pneumonia in Vietnam and other Asian countries was quite concerning [13,14]. These bacteria rapidly increased, which complicates initial antibiotic treatments, raises mortality rates, extends hospital stays, and drives up treatment costs, despite advancements in antibiotic therapies and improved supportive care. *K. pneumoniae* is presenting increasingly multi- and super-resistant, even to group 2 carbapenems [15], whereas *P. aeruginosa* is adept at developing new resistance mechanisms, which restricts treatment options, particularly, against colistin [16]. *A. baumannii* is a non-fermenting bacterium with a high infection rate and a rising incidence of carbapenem resistance [17]. Although *E. coli* exhibits a lower rate of resistance, it still represents a significant threat, especially with the emergence of strains that produce ESBL or carbapenemase [18]. Given the ever-evolving resistance patterns, it is crucial to monitor and keep the situation regarding pneumonia-causing bacteria updated for each geographic region and period.

Previous research has shown that bacteria are becoming increasingly resistant to antibiotics faster, more often, and at high levels [14,19,20]. Currently, gram-negative bacteria play a pathogenic role at a rate of around 70%, with common groups including the Enterobacteriaceae family (*E. coli, K. pneumoniae*), *A. baumannii*, and *P. aeruginosa* [21,22]. These bacteria can produce ESBL, which is resistant to all β-lactam antibiotics except carbapenems. However, some strains, such as New Deli Metalo-β-lactamase [23,24], can develop carbapenem-resistant carbapenemase. Many bacteria that cause hospital infections are multidrug-resistant; several strains of *A. baumannii* and *P. aeruginosa* are extended-drug–resistant or pan-drug–resistant (PDR) [25]. Currently, the situation of rising antibiotic resistance in bacteria is increasingly critical, with microorganisms causing pneumonia changing and increasing drug resistance significantly. According to studies conducted in Vietnam, the situation of growing antibiotic resistance in pneumonia-causing bacteria is quite worrisome [8,[26], [27], [28]].

For patients with severe pneumonia who require intensive care, the most important thing is to select the appropriate starting antibiotic for empirical treatment; therefore, understanding bacterial features and antibiotic resistance trends is critical. Furthermore, disease patterns and antibiotic resistance trends in bacteria might differ throughout countries, regions, geographic locations, and even hospitals and treatment departments. As a result, each location must have its own data on the prevalence and trend of antibiotic resistance. This study aimed to describe the features and antibiotic resistance trends of various gram-negative bacteria that cause CAP and HAP in Northern Vietnam from 2022 to 2024.

## Methods

### Patients and definitions

This study is a prospective observational, non-interventional study conducted from January 2022 to December 2024. All patients aged >18 years were diagnosed with bacterial pneumonia and received intensive care at the National Hospital for Tropical Diseases during the duration of the study. A total of 49 patients with CAP and 173 patients with HAP were selected based on the 2019 American Thoracic Society and the Infectious Diseases Society of America [29] and European Respiratory Society/European Society of Intensive Care Medicine/European Society of Clinical Microbiology and Infectious Diseases/Asociación Latinoamericana del Tórax [4] guidelines.

### Definition of pneumonia

• The American Thoracic Society and the Infectious Diseases Society of America 2019 guidelines define CAP as follows [29]:
- Pneumonia is contracted outside of a hospital or health care setting.
- Symptoms appear before to hospitalization or within 48 hours of admission.
- The presence of lower respiratory tract infection symptoms such as fever >38°C, cough, sputum production, difficulty breathing, muscle pain, joint pain, and pleuritic chest discomfort leads to the clinical diagnosis.
- The chest X-ray shows new infiltrates.
• The European Respiratory Society/European Society of Intensive Care Medicine/European Society of Clinical Microbiology and Infectious Diseases/Asociación Latinoamericana del Tórax guidelines describe HAP as follows [4]:
- Pneumonia after 48 hours of admission.
- Not in the incubation phase at admission.
The study’s pneumonia diagnostic criteria include:
- Clinical evidence (that meets two or more of the following criteria):
 + Symptoms include fever >37.8°C or hypothermia <36°C.
 + Cloudy or purulent sputum.
 + Leukocytosis >10,000 cells/µl or decline <4000 cells/µl.
 + Hypoxia (PaO_2_/FiO_2_ < 300).
- Radiological evidence: new or increasing infiltrates on the chest X-ray
- Microbiological evidence: determination of bacteria.

Criteria for patient selection in the study:

- Patient aged ≥18 years.
- Diagnosed with bacterial pneumonia as per the criteria described previously.
- Positive bacterial culture results from endotracheal aspirate or sputum.
- Must provide consent to participate in the study (or family consent if the patient is unable to sign due to being on mechanical ventilation).
- Received treatment in the intensive care unit for a minimum of 48 hours.
- For patients with CAP, samples must be taken at the time of admission or within the first 48 hours after admission.
- In cases where patients present multiple pneumonia episodes during their hospital stay, only the initial episode will be included in the study.

Exclusion criteria for the study include:

- Confirmed viral (including COVID-19), fungal, mycoplasma, or parasitic pneumonia.
- Patients with terminal cancer with a life expectancy of less than 3 months.
- Pregnant women.
- Negative culture results or mixed infection with unknown pathogens.
- Patients or families refused to participate in the study.
- Patients were transferred within 48 hours.
- Patients with HIV/AIDS.

### Definition of resistant bacteria

- Carbapenem-resistant microorganisms are resistant to at least one carbapenem.
- Quinolone-resistant bacteria are resistant to at least one quinolone (LVX, CIP).

#### Data collection

- Create a standardized study medical record form based on the Vietnamese Ministry of Health's guidelines and the study’s content.
- Collect demographic information, medical history, clinical symptoms at admission (for CAP) and at the commencement of HAP symptoms (for HAP), lesions on chest X-ray/computed tomography at each time point, microbiological results, and antibiotic susceptibility test results as soon as they are available. Clinicians decide on treatment regimens and monitor clinical developments. Document problems and clinical outcomes (recovery, death, length of hospital stays).
- The non-intervention concept requires researchers to observe and gather data without influencing treatment decisions. The clinical medical team makes all decisions on diagnosis and treatment based on accepted processes. Researchers do not provide recommendations about treatment regimens.

#### Culture, identification, and antibiotic susceptibility testing methods

Lower respiratory tract specimens (sputum, bronchial, alveolar, sputum trap) of patients with pneumonia were collected in adherence to standard procedures. Specimens from the CAP group of patients were collected at the time of admission. Specimens from the HAP group of patients were taken at the time of onset. The specimens were thereafter cultured on blood agar, MacConkey agar, and chocolate agar (Oxoid, England). Colonies growing on the agar plates were then separated and identified using VITEK 2 COMPACT (BioMérieux) and tested for antibiotic susceptibility using antibiotic diffusion discs.

Bacterial identification using VITEK 2 COMPACT and matrix-assisted laser desorption/ionization (MALDI)–time of flight (MALDI-TOF):

- VITEK 2 COMPACT: Dilute the pure cultured bacterial colony biomass in a tube of saline to a turbidity of 0.55-0.6 McFarland, then insert the identification card corresponding to the gram-negative and gram-positive bacteria. Insert the tube and identification card into the VITEK 2 COMPACT machine, then follow the manufacturer’s instructions. After 6-8 hours, the identification results will be analyzed and the name of the bacterial species will be provided.
- MALDI-TOF: Apply 1 µl of BTS Biotyper Standard solution on a MALDI target plate and spread each pure colony sample. Dry the sample at room temperature. To identify fungi or bacteria with thick shells and spore-forming cells, dry 1 µl of a formic acid solution at room temperature. Apply 1 µl of alpha-cyano-4-hydroxycinnamic acid matrix solution to each area, allow to dry at room temperature (5-20 minutes), then insert the MALDI target plate into the MALDI Biotyper (Bruker Microflex MALDI-TOF) and follow the manufacturer’s instructions for identification.

Antibiotic resistance test using the diffusion paper ring method (Kirby–Bauer): dilute the pure cultivated bacterial colony into a saline tube to a turbidity of 0.5 ± 0.05 McFarland. Dip a sterile cotton swab into the tube and apply it uniformly over the surface of the agar plate. Place the antibiotic paper rings (Oxoid, England) on top of the agar plate and incubate for 20-24 hours at 35-37°C, 5% CO_2_. Measure the zone of inhibition (including the antibiotic disc diameter in millimeters) and interpret the data using the Clinical and Laboratory Standards Institute guidelines (2022, 2023, and 2024 versions), which are updated annually and have three levels: S (sensitive), I (intermediate resistance), and R (resistant).

#### Data analysis

The data were analyzed using SPSS 22.0 software with descriptive statistics (percentage for qualitative variables and mean ± standard deviation for quantitative variables). The Pearson chi-square test was used to compare the CAP and HAP groups, as well as Fisher’s Exact test for qualitative variables. Student’s *t*-test was used for quantitative variables with a normal distribution. Statistical significance level: *P* <0.05.

#### Ethics

The research was conducted following ethical guidelines in medical studies and regulations regarding the protection of personal data. Approval was received from the Hospital Board of Directors and the Biomedical Research Ethics Council, under reference No. 30-2022/HĐĐĐ-NĐTƯ, which was signed on 2022.

## Results

The study included 222 patients with severe pneumonia who were treated in intensive care, with 49 cases of CAP and 173 cases of HAP meeting the selection criteria. The male to female ratio was roughly 2.9:1, and there was no difference between the CAP and HAP groups. The age group ≥65 years had the highest incidence of severe pneumonia (in the CAP and HAP groups with ages 42.9 and 47.4 years, respectively), with no difference in mean age between the CAP and HAP groups. Geographic distribution was similar between CAP and HAP, with ∼17% of patients living in the Northeast, Red River Delta (∼69%), Northwest (∼5%), and North Central (∼10%). Diabetes and dementia/paraplegia were more common in the HAP group (26.6% and 19.1%, respectively) than in the CAP group (16.3% and 14.3%, respectively), whereas liver disease was more prevalent in the CAP group (12.2%) than in the HAP group (6.4%). Other diseases occurred more frequently in the HAP group than in the CAP group (44.9% vs 14.5%). Patients with HAP showed higher quick Sepsis-related Organ Failure Assessment scores at levels 1 and 2 (35.3%, 49.1%) than CAP (22.4%, 38.8%) but lower scores at level 3 (10.4% vs 22.4%). Patients with HAP had a mean Charlson comorbidity index score of 1.23, which was greater than that of CAP (0.94). The mean treatment duration of patients with CAP was 14.04 days, which was shorter than that of the HAP group (22.98 days). However, the survival and mortality rates (treatment outcomes) in CAP and HAP were comparable (Table 1).

**Table 1:** Characteristics of patients with severe bacterial pneumonia treated in intensive care from 2022 to 2024 (n = 222).

Characteristics	Number of patients (n, %)	
	Community-acquired pneumonia	Hospital-acquired pneumonia
**Gender**		
Male	36 (73.5)	129 (74.6)
Female	13 (26.5)	44 (25.4)
**Age (years)**		
18-39	9 (18.4)	32 (18.5)
40-64	19 (38.8)	59 (34.1)
≥65	21 (42.9)	82 (47.4)
Mean ± SD	59.95±20.1	59.99±18.56
**Geographical distribution**		
Northeast	8 (16.3)	30 (17.3)
Red River Delta	33 (69.4)	117 (67.1)
Northwest	2 (4.1)	9 (5.2)
North Central	5 (10.2)	18 (10.4)
**Co-morbidity**		
Diabetes	8 (16.3)	46 (26.6)
Dementia/paraplegia	7 (14.3)	33 (19.1)
Liver disease	6 (12.2)	11 (6.4)
Heart/rheumatologic disease	2 (4.1)	20 (11.6)
Cancer	1 (2.1)	11 (6.4)
Chronic pulmonary disease	1 (2.1)	11 (6.4)
Renal disease	1 (2.1)	9 (5.2)
HIV/tuberculosis	1 (2.1)	7 (4.1)
Others	22 (44.9)	25 (14.5)
**Clinical severity score (qSofa)**		
0	8 (16.3)	9 (5.2)
1	11 (22.4)	61 (35.3)
2	19 (38.8)	85 (49.1)
3	11 (22.4)	18 (10.4)
**Charlson comorbidity index**		
0	29 (59.2)	83 (48)
1	6 (12.2)	26 (15)
2	8 (16.3)	34 (19.7)
3	1 (2.0)	16 (9.2)
4	5 (10.2)	9 (5.2)
6-9	0 (0.0)	5 (2.9)
Mean ± SD	0.94±1.38	1.23±1.58
**Treatment outcome**		
Survival	27 (69.2)	87 (70.7)
Death	12 (30.8)	36 (29.3)
**Length of hospital stay**		
Mean ± SD	14.04 ±13.35	22.98±18.32

qSofa, quick Sepsis-related Organ Failure Assessment.

A total of 222 lower respiratory tract specimens were collected from 222 patients, of which 194 (87.4%) isolated one type of bacteria and 28 (12.6%) isolated two types of bacteria, with patients with HAP having a higher rate of samples isolated two types of bacteria than the CAP group (14.5% vs 6.1%). In specimens isolated, two species of bacteria, *A. baumannii* + *K. pneumoniae* accounted for 53.6%, *A. baumannii* + *P. aeruginosa* for 28.6%, *K. pneumoniae* + *P. aeruginosa* for 7.1%, K. pneumoniae + *Proteus* for 3.6%, *S. aureus* + *K. pneumoniae* for 3.6%, and *S. aureus* + *Moraxella* for 3.6%. Only *A. baumannii* + *K. pneumoniae, K. pneumoniae* + *P. aeruginosa*, and *K. pneumoniae* + *Proteus* were found in patients with HAP. On the other hand, *S. aureus* + *K. pneumoniae* and *S. aureus* + *Moraxella*, were isolated exclusively in patients with CAP. The distribution of 250 bacterial strains recovered from 222 clinical samples revealed that *A. baumannii* (32.8%) had the highest proportion, followed by *P. aeruginosa* (23.6%), *K. pneumoniae* (20.4%), and S*. aureus* (8.0%), with other bacteria ranging from 0.4% to 3.2%. *A. baumannii* and *P. aeruginosa* were more prevalent in the HAP group (34.8% and 25.3%, respectively), but *S. aureus* (15.4%) was more prevalent in the CAP group (6.1%) (Table 2).

**Table 2:** Distribution of bacterial pathogens of pneumonia in patients treated in intensive care from 2022 to 2024 (n = 222).

Characteristics	Number of specimens (n, %)		Total (n, %)
	Community-acquired pneumonia	Hospital-acquired pneumonia	
**Specimen/number of bacteria (n = 222)**			
1 bacteria/specimen	46 (93.9)	148 (85.5)	194 (87.4)
2 bacteria/specimen	3 (6.1)	25 (14.5)	28 (12.6)
**Specimen with 2 bacteria (n = 28)**			
*A. baumannii + K. pneumoniae*	0 (0.0)	15 (60.0)	15 (53.6)
*A. baumannii + P. aeruginosa*	1 (33.3)	7 (28.0)	8 (28.6)
*K. pneumoniae + P. aeruginosa*	0 (0.0)	2 (8.0)	2 (7.1)
*K. pneumoniae + Proteus*	0 (0.0)	1 (4.0)	1 (3.6)
*S. aureus + K. pneumoniae*	1 (33.3)	0 (0.0)	1 (3.6)
*S. aureus + Moraxella*	1 (33.3)	0 (0.0)	1 (3.6)
**Distribution of bacteria (n = 250)**			
***Acinetobacter baumannii***	**13 (25.0)**	**69 (34.8)**	**82 (32.8)**
***Pseudomonas aeruginosa***	**9 (17.3)**	**50 (25.3)**	**59 (23.6)**
***Klebsiella pneumoniae***	**11 (21.2)**	**40 (20.2)**	**51 (20.4)**
*Staphylococcus aureus*	8 (15.4)	12 (6.1)	20 8.0)
*Stenotrophomonas maltophilia*	0 (0.0)	8 (4.0)	8 (3.2)
*Streptococcus pneumoniae*	4 (7.7)	2 (1)	6 (2.4)
*Escherichia coli*	1 (1.9)	3 (1.5)	4 (1.6)
*Burkholderia cepacia*	1 (1.9)	2 (1.0)	3 (1.2)
*Moraxella catarrhalis*	1 (1.9)	1 (0.5)	2 (0.8)
Corynebacterium striatum	0 (0.0)	2 (1.0)	2 (0.8)
*Burkholderia pseudomallei*	0 (0.0)	1 (0.5)	1 (0.4)
*Enterococcus faecalis*	1 (1.9)	0 (0.0)	1 (0.4)
*Others*^a^	3 (5.8)	8 (4.0)	11 (4.4)

a Others: *Enterobacter* spp., *Proteus* spp., *Burkholderia* spp., *Elizabethkingia* spp., *Chryseobacterium* spp., *Enterococcus* spp.*, Klebsiella* spp.*, Acinetobacter* spp*.*

**Figure 1:**
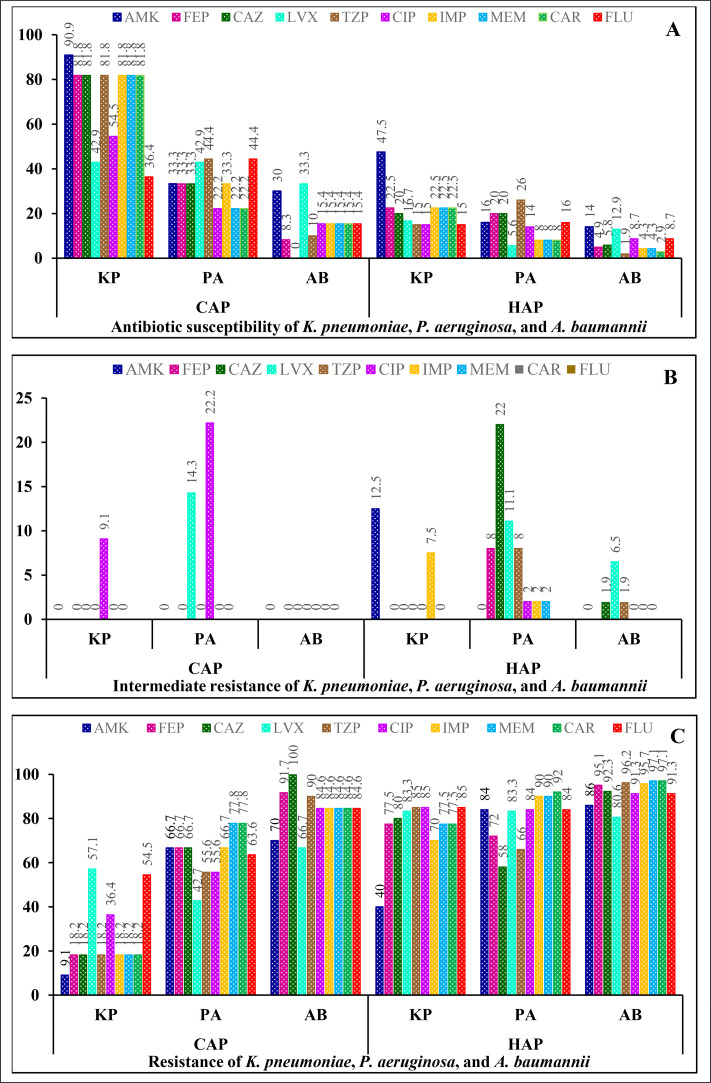
Antibiotic susceptibility (a), intermediate resistance (b), and resistance (c) levels of *A. baumannii, P. aeruginosa, K. pneumoniae* isolated from patients with CAP and HAP in the study. Note: AMK, amikacin; CAZ, ceftazidime; CAR, carbapenem (including imipenem, meropenem, and ertapenem); CAP, community-acquired pneumonia; CIP, ciprofloxacin; FEP, cefepime; FLU, fluoroquinolone (including levofloxacin and ciprofloxacin); HAP, hospital-acquired pneumonia; LVX, levofloxacin; TZP, piperacillin-tazobactam; IMP, imipenem; MEM, meropenem.

Three gram-negative bacteria, *A. baumannii, P. aeruginosa*, and *K. pneumoniae*, accounted for 76.8% of all bacterial infections causing pneumonia in patients treated in intensive care from 2022 to 2024. We analyzed the features and drug resistance rates of these three gram-negative bacteria. The rates of sensitivity, intermediate resistance, and resistance of bacteria depicted in Figure 1 were averaged across 3 years: 2022, 2023, and 2024.

The data comparing the sensitivity of *A. baumannii, P. aeruginosa*, and *K. pneumoniae* revealed that bacteria in the patients with community-acquired pneumonia (CAP) exhibited a higher rate of sensitivity to antibiotics than in the hospital-acquired pneumonia (HAP), with *K. pneumoniae* presented a sensitivity of 81.8-90.9% to amikacin (AMK), Cefepime (FEP), ceftazidime (CAZ), piperacillin-tazobactam (TZP), imipenem (IMP), meropenem (MEM), and carbapenem (CAR) and 36.4-54.5% to levofloxacin (LVX), ciprofloxacin (CIP), and fluoroquinolone (FLU). *A. baumannii* showed the lowest antibiotic sensitivity, ranging from 8.3% to 33.3%, with 100% resistance to CAZ. In addition, bacteria isolated from patients with HAP presented a far lower rate of antibiotic sensitivity than CAP bacteria, with *K. pneumoniae* only sensitive from 15.0% to 47.5%, *P. aeruginosa* from 8.0% to 26.0%), and *A. baumannii* from 1.9% to 12.9% (Figure 1a).

*A. baumannii, P. aeruginosa*, and *K. pneumoniae* demonstrated very low intermediate resistance to antibiotics in the patients with CAP and HAP, with slightly difference between the two groups. They only showed low intermediate resistance to several antibiotics, including LVX (6.5-14.3%), IMP (2.0-7.5%), AMK (12.5%), and CIP (9.1-22.2%) (Figure 1b).

Comparing the antibiotic resistance rates among *A. baumannii, P. aeruginosa*, and *K. pneumoniae*, it was revealed that bacteria in the patients with CAP exhibited lower resistance levels than those in the patients with HAP. Notably, *K. pneumoniae* within the CAP group showed resistance levels ranging from 9.1% to 54.5% against the antibiotics tested, followed by *P. aeruginosa*, with resistance rates of 42.7-77.8%, whereas *A. baumannii* exhibited the highest resistance levels, varying from 66.7% to 100.0%. In contrast, bacteria from the HAP group displayed higher resistance proportion, with *K. pneumoniae* shown rates from 40.0% to 85.0%, *P. aeruginosa* from 58.0% to 92.0%, and *A. baumannii* presented the highest resistance levels from 80.6% to 97.1% (Figure 1c).

We conducted a separate analysis of antibiotic resistance rates for CAP and HAP bacteria in the years 2022, 2023, and 2024 to examine how antibiotic resistance has changed over time.

**Figure 2:**
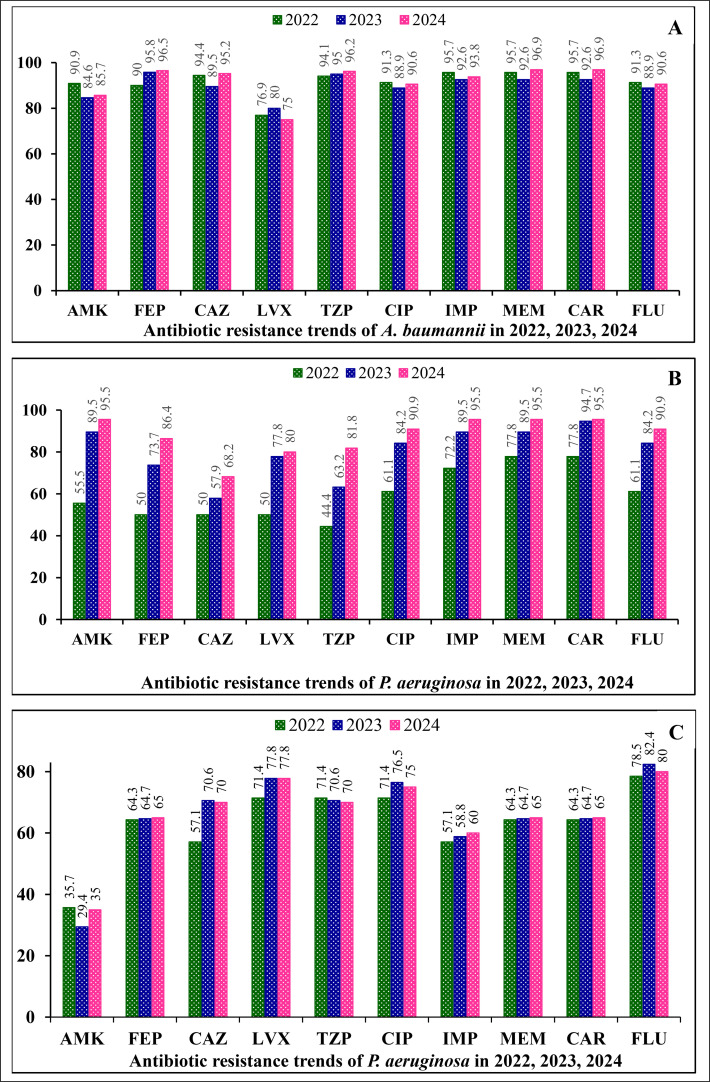
Comparison of antibiotic resistance levels of *A. baumannii* (a)*, P. aeruginosa* (b)*, K. pneumoniae* (c) strains in 2022, 2023, and 2024. Note: AMK, amikacin; CAR, carbapenem; CAP, community-acquired pneumonia; CAZ, ceftazidime; CIP, ciprofloxacin; FEP, cefepime; FLU, fluoroquinolone; HAP, hospital-acquired pneumonia; LVX, levofloxacin; IMP, imipenem; MEM, meropenem; TZP, piperacillin-tazobactam.

The findings revealed that *A. baumannii* exhibited minimal fluctuations in antibiotic resistance rates across the years 2022, 2023, and 2024. The resistance to LVX was at the lowest level (76.9% in 2022, 80.0% in 2023, and 75.0% in 2024), whereas other antibiotics, including AMK, FEP, CAZ, TZP, CIP, IMP, MEM, CAR, and FLU, showed negligible variations in resistance levels between 2022, 2023, and 2024 (ranging from 1.0% to 4.5%) (Figure 2a).

*P. aeruginosa* antibiotic resistance increased significantly over time, with resistance to AMK, FEP, CAZ, LVX, TZP, CIP, IMP, MEM, CAR, and FLU ranging from 44.4% to 77.8% by 2022. This rose to 57.9-94.7% (an average rise of approximately 20.4%) in 2023 and 68.2-95.5% in 2024 (an average increase of approximately 7.6%) (Figure 2b).

Antibiotic resistance in *K. pneumoniae* slightly changed between 2022, 2023, and 2024, with the lowest resistance rate compared with *A. baumannii* and *P. aeruginosa*. Resistance to AMK was 29.4-35.7%, followed by IMP (57.1-60.0%), MEM, and CAR (64.3-65.0%), with the highest resistance to LVX (71.4-77.8%) (Figure 2c).

## Discussion

This study is one of the few that examines the characteristics and trends in antibiotic resistance of gram-negative bacteria responsible for CAP and HAP in patients in intensive care in Vietnam. The findings indicated that men are approximately 2.8 times more likely to develop CAP and three times more likely to experience HAP than women, a ratio significantly higher than that reported by Millet et al. (1.8 times) [30], Arnold et al. (1.5 times) [31], Riverco-Callle et al. (1.2 times) [32], Pessoa et al. (1.2 times) [33], and de Miguel-Yanes et al. (1.4 times) [34]. The variations in the male to female ratio of pneumonia among different studies highlight disparities in physiology, underlying health conditions or exposure to risk factors, geographic variations, and lifestyle differences between the sexes. Several additional studies have also indicated that men face a greater risk of pneumonia due to higher prevalence of smoking, chronic obstructive pulmonary disease, and other health issues related to respiratory difficulties [[35], [36], [37]]. This underscores the need for tailored infection control and prevention strategies for each gender to lessen the risk of pneumonia.

The majority of patients diagnosed with pneumonia were aged >60 years (65%), which aligns with findings that severe infections are more prevalent among older adults due to factors such as immunosuppression, chronic health issues, and extended hospital stays. Elderly people frequently experience a compromised immune system, diminished capacity to expel secretions, and reduced effectiveness of their inflammatory responses, increasing their vulnerability to severe infections. Numerous previous studies have indicated a correlation between advancing age and elevated mortality rates from pneumonia [[38], [39], [40]], highlighting the necessity for rigorous infection control measures and effective treatment strategies for this demographic. Therefore, it is crucial to closely monitor the infection status, optimize nutritional intake, and implement preventive strategies—such as effective glycemic management and upkeep of respiratory function—in the care of elderly patients in the intensive care unit to diminish the likelihood of acquiring nosocomial pneumonia.

In this study, the incidence of polymicrobial pneumonia was found to be 12.6%, whereas monomicrobial pneumonia was observed at a rate of 87.3%. The microbiological culture results revealed that three gram-negative bacteria—*A. baumannii, P. aeruginosa*, and *K. pneumoniae*—represented a significant proportion among patients with CAP and HAP. This highlights a substantial risk of antibiotic resistance with these bacteria, which develop mechanisms and rates of resistance more rapidly and effectively than other bacteria, particularly, those associated with HAP. Some studies indicate that bacteria responsible for hospital-acquired infections in intensive care units exhibit low levels of antibiotic sensitivity. The comparison of antibiotic susceptibility between the CAP and HAP groups in this research indicated that the HAP group exhibited a significantly lower sensitivity rate compared with the CAP group. This highlights the considerable selective pressure from antibiotics and the immunocompromised condition of hospitalized patients. Such a scenario presents challenges in selecting appropriate antibiotics, including the need to use multiple agents to achieve the required therapeutic coverage, the potential for drug interactions and heightened drug toxicity, and the challenges associated with monitoring treatment efficacy. Consequently, it is essential to consider alternative treatment regimens and optimize dosage and administration based on pharmacokinetics and pharmacodynamics or investigate combination antibiotic therapies informed by pharmacokinetics and pharmacodynamics principles. Furthermore, the option of using aminoglycoside antibiotics (e.g. tobramycin and gentamicin) for treating gram-negative infections should be considered if dosage optimization is achieved through therapeutic monitoring by measuring drug levels in the bloodstream.

The study observed generally elevated rates of multidrug resistance and super antibiotic resistance among bacteria, particularly, *P. aeruginosa* and *A. baumannii*. Regarding the upward trend in antibiotic resistance, the resistance rates of *A. baumannii* and *K. pneumoniae* displayed no significant differences between the years 2022, 2023, and 2024, suggesting that the resistance levels for these bacteria have reached a plateau. This presents a considerable challenge for infection control and prevention because they are typically susceptible to only a limited number of antibiotics, mainly, colistin and certain specialized regimens. In this research, we did not encounter any colistin-resistant bacteria, which makes this antibiotic a common consideration for treating multidrug-resistant infections. However, it is essential to assess the potential side effects (nephrotoxicity) associated with colistin use. On the other hand, a rise in multidrug-resistant and PDR strains of *P. aeruginosa* is anticipated for 2023 and 2024, indicating that these bacteria are developing new resistance mechanisms, including the production of ESBL, reduced permeability of the outer membrane, and enhanced efflux pumps that expel antibiotics from within the cell. These results highlight concerns about the spread of PDR and multidrug-resistant strains in health care facilities and general communities. The presence of PDR is a serious threat because it leaves no effective antibiotic treatment options available, which can result in increased mortality rates and extended hospitalizations. Therefore, it is crucial to reinforce infection control measures, which include vigilant monitoring of antibiotic usage and the execution of infection prevention strategies.

In addition, it is crucial to track trends in antibiotic resistance over time and recognize the risk factors linked to heightened antibiotic resistance. Previous research has indicated that extended use of carbapenem antibiotics correlates with a higher risk of developing carbapenem resistance in gram-negative bacteria, particularly, *A. baumannii, K. pneumoniae*, and *P. aeruginosa*. This underscores the necessity for tailored treatment plans based on clinical microbiology findings and ongoing antibiotic resistance surveillance to adjust strategies effectively. The findings from this study highlight the seriousness of antibiotic resistance in CAP and HAP, particularly, among gram-negative bacteria such as *A. baumannii, K. pneumoniae*, and *P. aeruginosa*. The rise in antibiotic resistance underscores the urgent need for ongoing monitoring and stricter infection control practices within health care settings. These actions will assist in curbing the spread of drug-resistant bacteria and enhance the effectiveness of treatments for patients.

In conclusion, gram-negative bacteria responsible for CAP and HAP in Northern Vietnam represent a significant proportion (>70%), particularly, *A. baumannii* and *P. aeruginosa* in patients with CAP and *A. baumannii, K. pneumoniae*, and *P. aeruginosa* in patients with HAP, which show substantial resistance to various aminoglycosides, cephalosporins, carbapenems, and fluoroquinolones. The antibiotic resistance rates of *A. baumannii* and *K. pneumoniae* have remained relatively stable from 2022 to 2024; however, there is a notable increase in the resistance rates of P*. aeruginosa* in 2023 and 2024. It is essential to monitor the fluctuations in resistance rates and trends among gram-negative bacteria closely.

## Funding

This research did not receive any specific grant from funding agencies in the public, commercial, or not-for-profit sectors.

## Ethical approval

The ethical approval of this study was waived according to our center’s policy.

## Author contributions

Nguyen Quoc Phuong: study design, data collection, data analysis, manuscript writing, and revising. Tran Van Giang: study design, data collection, data analysis, manuscript writing, and revising. Pham Ngoc Thach: data analysis, writing draft.

## Declaration of competing interest

The authors have no competing interests to declare.
